# Regulatory Effect of *Ficus carica* Latex on Cell Cycle Progression in Human Papillomavirus-Positive Cervical Cancer Cell Lines: Insights from Gene Expression Analysis

**DOI:** 10.3390/ph16121723

**Published:** 2023-12-12

**Authors:** Muharrem Okan Cakir, Ugur Bilge, Arshia Ghanbari, G. Hossein Ashrafi

**Affiliations:** 1 School of Life Sciences, Pharmacy and Chemistry, Kingston University London, London KT1 2EE, UK; m.okan@kingston.ac.uk (M.O.C.); k1508136@kingston.ac.uk (A.G.); 2 Department of Biostatistics and Medical Informatics, Faculty of Medicine, Akdeniz University, Antalya 07050, Turkey; ubilge@akdeniz.edu.tr

**Keywords:** HPV-positive, cervical cancer, fig latex, *Ficus carica*, RNA-Seq, high risk HPV

## Abstract

Cervical cancer presents a significant global health concern with high-risk human papillomaviruses (HPVs) identified as the main cause of this cancer. Although current treatment methods for cervical cancer can eliminate lesions, preventing metastatic spread and minimizing tissue damage remain a major challenge. Therefore, the development of a safer and innovative therapeutic approach is of the utmost importance. Natural products like fig latex, derived from the *Ficus carica* tree, have demonstrated promising anti-cancer properties when tested on cervical cancer cell lines. However, the specific mechanisms by which fig latex exerts its effects are still unknown. In this study, we conducted RNA-Seq analysis to explore how fig latex may counteract carcinogenesis in HPV-positive cervical cancer cell lines, namely, CaSki (HPV type 16-positive) and HeLa (HPV type 18-positive). Our results from this investigation indicate that fig latex influences the expression of genes associated with the development and progression of cervical cancer, including pathways related to “Nonsense-Mediated Decay (NMD)”, “Cell Cycle regulation”, “Transcriptional Regulation by TP53”, and “Apoptotic Process”. This selective impact of fig latex on cancer-related pathways suggests a potential novel therapeutic approach for HPV-related cervical cancer.

## 1. Introduction

Cervical cancer is a prevalent malignancy in women with a staggering incidence of 604,127 new cases and 341,831 deaths globally reported in 2020 [1]. Among the myriad factors contributing to the development and progression of this disease, infection by high-risk human papillomaviruses (HPVs) emerges as a key player [2]. Although the majority of HPV infections are resolved by the host immune system within several months of exposure, viral lesions persist and gradually escalate into cancerous growths in certain cases [3,4]. High-risk HPV strains, particularly types 16 and 18, play a significant role in driving the development of cervical cancer [5,6]. Their oncoproteins, E6 and E7, are key factors in orchestrating the intricate process of cellular transformation into a malignant state [6]. Specifically, the E6 oncoprotein undermines the function of the tumour suppressor protein P53, impairing its role in orchestrating apoptosis and contributing to the evasion of cell death [7,8,9,10,11]. In parallel, E7 disrupts the interaction between the retinoblastoma (Rb) protein and the transcription factor E2F, tilting the G1/S cell cycle transition off balance and fostering uncontrolled proliferation [12,13,14].

Despite the availability of preventive HPV vaccines and advances in screening and early detection, the clinical landscape of advanced cervical cancer treatment remains ensnared in challenges largely driven by the complex interplay of HPV oncoproteins [5,15,16]. While conventional therapeutic modalities like surgical intervention, radiation therapy, and chemotherapy play pivotal roles, their utility is marred by a spectrum of unfavourable side effects [17]. Therefore, there is a growing demand for new drugs to improve the treatment of cervical cancer, particularly for patients with advanced disease who have failed standard therapies [18,19].

Recently, natural compounds have garnered substantial scientific interest for their potential therapeutic efficacy against various cancer types [20,21]. These compounds are of particular interest due to their ability to induce apoptosis, inhibit cellular proliferation and modulate crucial cellular signalling pathways [22]. Within this context, fig latex, extracted from *F. carica* species, stands out due to its compelling potential anticancer properties. Recent studies have shed light on the cytotoxic effects of fig latex on a wide range of cancer cell lines including those associated with cervical, gastric, and colorectal cancers [23,24,25]. Importantly, our previous research has shown that fig latex suppressed cervical cancer cell growth and induced apoptosis by downregulating the E6 and E7 oncoproteins while upregulating the tumour suppressor proteins P53 and Rb. These findings firmly establish fig latex as a promising candidate for cancer therapy [25].

Based on the earlier findings above, we conducted an in-depth analysis of fig latex on genes related to cellular growth in HPV-positive cervical cancer cell lines by using RNA sequencing (RNA-Seq). Our results clearly demonstrated that fig latex suppressed the growth in HPV-positive cervical cancer cell lines, specifically CaSki and HeLa, without any observed cytotoxic effects on normal/non-cancerous cervical cells (HCKT1). The analysis of RNA-Seq data unveiled fig latex’s regulatory role in the expression of genes crucial to various cancer related pathways, particularly those involved in “Nonsense Mediated Decay (NMD)”, “Cell Cycle”, “Transcriptional Regulation by TP53”, and “Apoptotic Process”. This selective impact of fig latex on cancer-related pathways suggests a potential novel therapeutic approach for HPV-related cervical cancer.

## 2. Results

### 2.1. Fig Latex Inhibits the Growth of HPV-Positive Cervical Cancer Cell Lines

**Figure 1:**
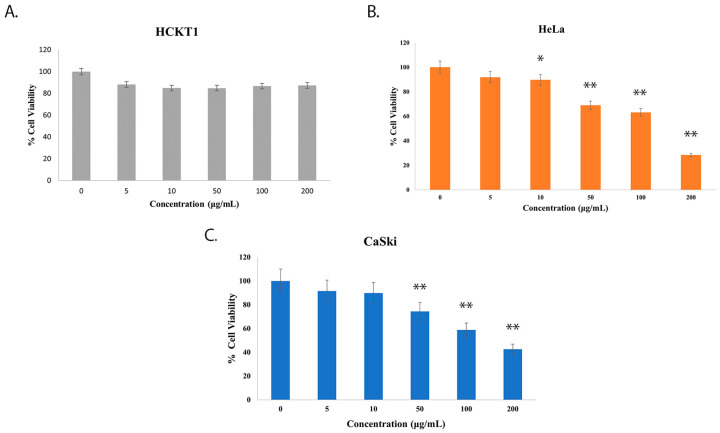
The effect of whole fig latex on the growth of cervical cell lines. (**A**) HCKT1, (**B**) HeLa, and (**C**) CaSki were treated with different concentrations of whole fig latex (5 μg/mL, 10 μg/mL, 50 μg/mL, 100 μg/mL, and 200 μg/mL) for 72 h. The SRB assay was used to determine cell viability. Data points represent the mean ± SD of three independent experiments, each performed in triplicate. The IC50 values were calculated using R software (version 3.6.1) using a sigmoidal curve fit based on nonlinear regression. Statistical significance was assessed by one-way ANOVA followed by Tukey post hoc test and represented as follows: * *p* < 0.05 and ** *p* < 0.01 vs. whole fig latex 0 μg/mL in DMSO. Doxorubicin was employed as the positive control in the SRB assay.

To explore the effect of fig latex on the growth of distinct cervical cell lines, we conducted experiments using three cell types: normal human cervical keratinocytes (HCKT1) and two HPV-positive cervical cancer cell lines (HeLa HPV18+ and CaSki HPV16+). These cells were subjected to varying concentrations of fig latex (5 μg/mL, 10 μg/mL, 50 μg/mL, 100 μg/mL, and 200 μg/mL) for a duration of 72 h. The viability of these cells was assessed using the Sulforhodamine B (SRB) colorimetric assay. Notably, fig latex demonstrated no cytotoxic effects on normal human cervical keratinocytes when contrasted with cervical cancer cells. Remarkably, the IC50 values for fig latex after 72 h on HeLa and CaSki cells were determined to be 106 μg/mL and 110 μg/mL, respectively (Figure 1B,C). These observations underline the potential of specific fig latex concentrations to selectively induce cytotoxicity in HPV-positive cervical cancer cells without any adverse effect on normal cells (Figure 1A).

### 2.2. Fig Latex Induces Cell Cycle Arrest at Sub G1 in HPV-Positive Cervical Cancer Cell Lines

**Figure 2:**
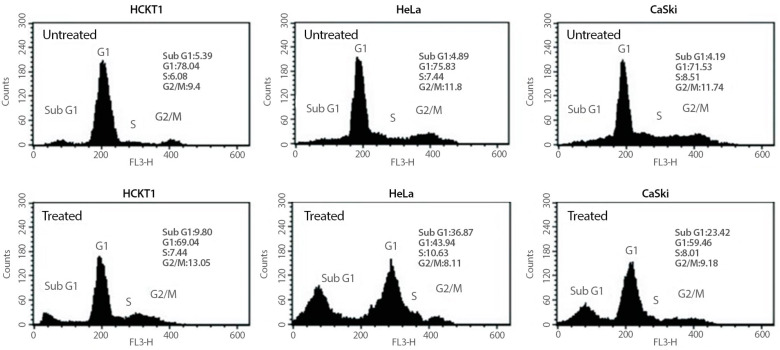
The effect of fig latex on the cell cycle progression in human cervical cell lines. Different human cervical cancer cells, HeLa and CaSki, and normal healthy human cervical keratinocytes were incubated with 100 μg/mL of fig latex for 72 h. The representative cell cycle distribution of each cell type was analysed using flow cytometry. All experiments were conducted three times independently, each in triplicate.

To investigate whether the growth inhibitory effect of fig latex on HeLa and CaSki cells is related to the cell cycle arrest, changes in cell cycle progression of those cancer cells were determined after 100 μg/mL of fig latex treatment using flow cytometry. As shown in Figure 2, cancer cells in sub-G1 phase were increased under fig latex treatment. This suggests that fig latex inhibited the cellular proliferation of human cervical cancer cell lines HeLa and CaSki via the arrested G1 phase of the cell cycle. In contrast, HCKT1 cells treated with fig latex showed approximately the same ratio in all phases of the cell cycle.

### 2.3. Transcriptomic Profiling of Different HPV-Positive Cervical Cancer Cells upon Whole Fig Latex Treatment

To investigate the gene expression profile of HPV-positive human cervical cancer cells upon fig latex treatment, the cells, HeLa (HPV18-positive) and CaSki (HPV16-positive), were subjected to treatment with 100 μg/mL of fig latex that was a concentration closely aligned with the IC50 value. We performed RNA sequencing (RNA-Seq) to decipher the changes in the gene expression profiles of human cervical cancer cells upon treatment.

The subsequent analysis of differential gene expression uncovered a total of 149 genes that exhibited significant differences in expression levels within the HPV-positive cancer cell lines. Among these genes, 65 demonstrated consistent downregulation, while 84 displayed consistent upregulation upon exposure to fig latex treatment. This dynamic shift in gene expression profiles paints a comprehensive picture of the genetic responses triggered in HPV-positive cervical cancer cell lines following exposure to fig latex (Table S1).

### 2.4. Analysis of Differentially Expressed Genes in HPV-Positive Cervical Cancer Cell Lines upon Fig Latex Treatment Using KEGG Pathway Enrichment Analysis

Pathway enrichment analysis was employed to scrutinize the effects of fig latex treatment on differentially expressed genes within HPV-positive cervical cancer cell lines. The results indicated that the genes modulated by fig latex treatment were crucial for several cell processes including “Nonsense Mediated Decay (NMD)”, “Cell Cycle”, “Transcriptional Regulation by TP53”, and “Apoptotic Process” (Table 1). Noteworthily, a cluster of genes overlapped across these pathways, implying their indispensable role in the regulation of the cell cycle, mRNA stability, and programmed cell death (apoptosis). These collective findings suggest that fig latex holds the potential to regulate pivotal signalling cascades, intricately linked to cell growth and genomic instability, thereby extending its potential therapeutic significance to the management of HPV-associated cervical cancers.

### 2.5. Investigation of Differentially Expressed Genes in HPV-Positive Cervical Cancer Cell Lines upon Fig Latex Treatment via Kinase Enrichment and Chromatin Enrichment Analysis

**Figure 3:**
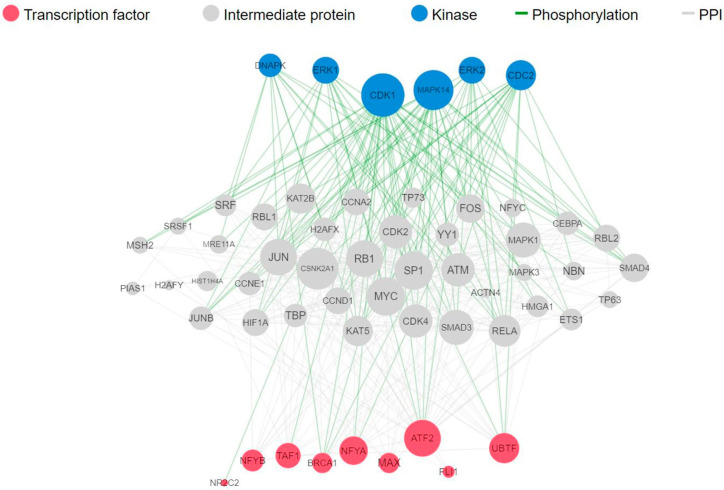
Complete upstream pathways that connect enriched transcription factors to kinases through known protein–protein interactions. Red nodes represent top transcription factors predicted to regulate the expression of the input gene list; grey nodes represent proteins that physically interact with the enriched transcription factors and connect them. Blue nodes represent the top predicted protein kinases known to phosphorylate the proteins within the expanded subnetwork. Green network edges/links represent kinase–substrate phosphorylation interactions between kinases and their substrates, while grey network edges represent physical protein–protein interactions.

To identify the transcription factors and kinases that regulate downregulated genes upon fig latex treatment, we employed in silico methodologies, including Chromatin Enrichment Analysis (CheA) and Kinase Enrichment Analysis (KEA). Our analysis underscores the prominence of transcription factors such as NFYB, NFYA, MAX, and TAF1, alongside kinases CDK4, CDK1, and MAPK1 (Table 2A,B). These molecules play pivotal roles in orchestrating the observed gene expression changes induced by fig latex, illuminating the complex interplay between the fig latex treatment and cellular regulatory networks. Furthermore, a comprehensive upstream pathway analysis of enriched transcription factors and kinases revealed that fig latex treatment impacts key proteins involved in cell cycle regulation. Specifically, the regulation of MAPK1, CDK2, and MYC proteins, which are crucial for cell growth, by enriched transcription factors was observed (Figure 3). The discovery of these key transcription factors and regulatory kinases as the regulators of downregulated genes in the cell cycle reveals that fig latex holds potential for combatting HPV-positive cancers at the molecular level.

## 3. Discussion

Cervical cancer, primarily instigated by high-risk human papillomavirus (HPV) infection, poses a substantial global health burden [1,17]. A pivotal challenge in combating cervical cancer is rooted in the tumour cell’s aggressive growth, primarily orchestrated by HPV oncoproteins E6 and E7 [16]. These oncoproteins play a pivotal role in overriding cellular control mechanisms, leading to carcinogenesis [26]. This not only leads to the progression of the cancer but also contributes to resistance against current therapies, resulting in poor clinical outcomes [18,27]. Consequently, there is a pressing need to develop novel therapeutic strategies [17]. The realm of natural products has emerged as a promising area of interest in cancer research due to their potential anticancer activity [22]. Our previous investigation highlighted the therapeutic potential of *F. carica* L., a natural product derived from figs, in the context of HPV-positive cervical cancer cell lines [25]. That study also provided novel insights into the chemical composition of crude fig latex through NMR/MS analysis, suggesting that the active components are likely to be lipophilic, possibly derivatives of chlorogenic, ferulic, or caffeic acid combined with a plant sterol [25]. However, the precise molecular mechanisms through which fig latex impacts HPV-positive cervical cancer cells, particularly its ability to counteract the carcinogenic effects mediated by HPV oncoproteins E6 and E7, remain to be elucidated. In the light of this, our current study aimed to unravel the potential of fig latex as a therapeutic approach to counteract the effect of HPV oncoproteins E6 and E7 in cervical carcinogenesis. 

To address this objective, we treated HPV-positive cervical cancer cells with fig latex and investigated the cellular growth-related gene expression patterns and signalling pathways. Through an extensive RNA-Seq analysis, we meticulously scrutinised the transcriptome of fig-latex-treated cells in comparison to the control group. This methodology allowed us to pinpoint genes with varying expression levels and provided insights into the precise molecular modifications triggered by fig latex treatment. 

Our investigation showed the growth inhibitory effects of fig latex on HPV-positive cervical cancer cells, in agreement with our earlier observations [25]. The treatment with fig latex resulted in a significant inhibition of cell growth in HeLa and CaSki, with calculated IC50 values of 106 and 110 μg/mL, respectively. Importantly, the treatment with fig latex had no discernible effect on normal human cervical keratinocytes (HCKT1), indicating the selective cytotoxicity of fig latex toward HPV-positive cervical cancer cells. Furthermore, our investigation looked into the cell cycle dynamics, revealing intriguing outcomes. The data from this analysis indicated that fig latex induces cell death in sub G1 phase in both HeLa and CaSki cells. The sub G1 phase is typically associated with cells undergoing apoptosis, at which fragmented DNA accumulates, distinguishing apoptotic and necrotic cells from the cell cycle [28]. Apoptosis plays a critical role in eliminating aberrant cells, and the induction of sub G1 phase arrest suggests that fig latex treatment may activate apoptotic pathways, a vital aspect in combating cancer progression [29,30,31]. These findings further emphasize the potential of fig latex to disrupt viral cell cycle checkpoints, thereby contributing to its selective cytotoxic effect on cervical cancer cells.

Beyond the growth inhibitory effect of fig latex, this study also reports that fig latex exhibits selective cytotoxicity against HPV-positive cervical cancer cells, by orchestrating intricate signalling pathways related to cervical cancer development and progression. These pathways are the Nonsense Mediated Decay (NMD) pathway (*p*-value: 2.21 × 10^−7^), the Cell Cycle pathway (*p*-value: 3.02 × 10^−3^), and the Transcriptional regulation by P53 pathway (*p*-value: 3.42 × 10^−5^). The NMD pathway is specifically known for its role in degrading transcripts harbouring premature termination codons (PTCs) [32,33,34,35]. Notably, several key genes of this pathway including *RPS27A*, *RNF111*, and *RPS6* were consistently upregulated with fig latex treatment. Those key genes in the NMD pathway are involved in crucial steps of mRNA degradation and surveillance. *RPS27A* encodes a ribosomal protein involved in mRNA surveillance and ribosome quality control mechanisms [36,37]. *RNF111*, also known as Arkadia, functions as an E3 ubiquitin ligase, influencing mRNA stability and processing [35]. *RPS6*, a ribosomal protein, participates in the translation of specific mRNAs, regulating their stability and turnover [38]. HPV infection has been implicated in perturbing normal gene expression by promoting the degradation of specific cellular transcripts [39]. In particular, HPV E6 and E7 oncoproteins can disrupt normal cellular processes, including the regulation of mRNA stability [40]. In line with this, recent studies have demonstrated that the NMD pathway can target and degrade viral oncogenes, thereby exhibiting tumour suppressive properties [41,42,43]. These findings suggest that by activating the NMD pathway, fig latex may contribute to reducing the expression of HPV oncogenes, thereby suppressing their tumorigenic potential.

Secondly, our analysis of HPV-positive cancer cell lines treated with fig latex showed a significant enrichment of the Cell Cycle pathway, indicating a potential link between fig latex and cellular growth. Key genes involved in cell cycle regulation were downregulated, including *PCNA*, *POLD3*, *PRIM1*, and *ORC2*. *PCNA* is a vital component for DNA replication and repair processes, crucial for cell proliferation [44]. *POLD3* encodes a subunit of DNA polymerase delta, responsible for DNA synthesis during the S phase of the cell cycle [45,46]. *PRIM1* is involved in initiating DNA synthesis and maintaining genomic stability, while *ORC2* plays a crucial role in DNA replication initiation [47,48,49]. These key genes are intricately involved in the DNA replication and repair mechanisms that are often subverted or disrupted by HPV oncoproteins during the viral life cycle within cervical cells [15]. The dysregulation of the cell cycle is a hallmark of cancer, including HPV-associated cervical cancer [50]. HPV oncoproteins, particularly E6 and E7, disrupt the normal cell cycle control mechanisms, promoting uncontrolled proliferation and genomic instability [51,52,53]. Our findings suggest that fig latex treatment may inactivate the Cell Cycle pathway in HPV-positive cervical cancer cells. These findings are also consistent with our previous studies, where fig latex treatment was shown to inhibit the expression of HPV E6 and E7 oncoproteins, thereby leading to the suppression of cancer cell growth [25].

Thirdly, our pathway enrichment analysis of RNA-Seq data from HPV-positive cancer cell lines treated with fig latex revealed a significant enrichment of the Transcriptional Regulation by TP53 pathway (*p*-value: 3.42 × 10^−5^). This finding unveils a potential connection between fig latex and anti-cancer response by highlighting the upregulation of key genes, including *RAD1* and *YWHAQ*. *RAD1* is a component of the *RAD9-HUS1-RAD1* complex, contributing to DNA repair and cell cycle checkpoint activation [54]. *YWHAQ*, part of the 14-3-3 protein family, participates in regulating various cellular processes including cell cycle control, apoptosis, and DNA damage response [55]. These genes are known to play critical roles in cellular processes that intersect with HPV-associated molecular mechanisms, particularly in DNA repair and cell cycle regulation, which are often disrupted by HPV oncoproteins in cervical cancer progression [56]. The Transcriptional Regulation by TP53 pathway is essential for the control of cell cycle arrest, DNA repair, apoptosis, and senescence [57,58]. The *TP53* gene, also known as *p53*, is a tumour suppressor that plays a crucial role in maintaining genomic stability and preventing the development of cancer [59]. In HPV-associated cervical cancer, the HPV E6 oncoprotein promotes the degradation of p53, leading to the dysregulation of TP53-dependent transcriptional regulation [8]. Moreover, it is worth noting that our previous study demonstrated that fig latex treatment rescued the activity of P53 [25]. In line with this, by inhibiting E6-mediated p53 degradation, fig latex treatment may contribute to the restoration of TP53 transcriptional regulation in HPV-positive cervical cancer cells.

Lastly, our comprehensive analysis of the Apoptotic Process pathway after fig latex treatment in HPV-positive cancer cell lines exhibited a notable enrichment (*p*-value: 1.06 × 10^−4^), indicative of an intricate modulation of genes pivotal in cellular apoptosis [60,61]. This enrichment significantly correlates with the sub G1 arrest observed in both HPV-positive cervical cancer cell lines upon fig latex treatment. The activation of apoptotic genes like *PDCD6*, associated with apoptotic signalling cascades, and *DDRGK1*, implicated in DNA repair and apoptosis, suggests that fig latex triggers apoptosis pathways, leading to cell death specifically in HPV-positive cancer cells [62,63]. This substantial induction of apoptotic mechanisms, coupled with the observed sub G1 arrest, holds promising therapeutic implications. Fig latex, by selectively inducing apoptosis in HPV-positive cancer cells while sparing normal cells, could emerge as an effective strategy for targeted treatment in cervical cancer associated with HPV infection.

To uncover further molecular networks modulated by fig latex, transcription factors and kinase enrichment analysis was performed. The identification of prominent transcription factors (NFYB and NFYA) and regulatory kinases (CDK4, CDK1, and MAPK1) that govern the expression of downregulated genes following fig latex treatment highlights the potential of this natural remedy for targeting and modulating key players in cancer progression [64,65,66,67,68,69]. Particularly, our upstream pathway analysis reveals the impact of fig latex on essential proteins involved in cell cycle regulation, including MAPK1, CDK2, and MYC, which are integral to cell growth [64,65,66,67,68,69,70,71,72]. These findings signify fig latex’s potential in disrupting critical pathways associated with cancer cell proliferation. The ability to influence such regulatory networks at the molecular level holds promise for the development of novel, precision-driven therapies for HPV-positive cancers. 

Furthermore, our investigation demonstrated substantial cell cycle arrest and apoptosis induced by fig latex treatment in HPV-positive cervical cancer cells. The observed effects, specifically the cell cycle arrest and apoptosis, are likely attributed to the constituents present in fig latex, as identified in our previous study [25]. Recent research supports the potential roles of these components in eliciting such responses in cancer cells. For instance, chlorogenic acid derivatives, one of the identified components, have been associated with apoptotic effects by regulating apoptotic pathways [73,74,75]. Additionally, ferulic and caffeic acid derivatives, recognized for their antioxidant properties, have demonstrated apoptotic induction and potential interference with cell cycle progression [76,77,78]. Plant sterols have been linked to modulating apoptotic pathways and cell cycle regulation [79,80,81]. These findings from recent research align with our observed outcomes, suggesting that the identified components within fig latex may contribute to its ability to induce cell cycle arrest and apoptosis in HPV-positive cervical cancer cells.

In conclusion, our findings provide valuable insights into the molecular mechanisms underlying fig latex’s action and its potential in combating HPV-positive cervical cancer by inhibiting cancer cell growth. By targeting key pathways associated with cell proliferation, fig latex and its active components hold promise for the development of novel therapeutic strategies against cervical cancer. 

## 4. Materials and Methods

### 4.1. Chemicals and Reagents

Cell Culture medium, Dulbecco’s modified Eagle medium (DMEM), and Keratinocyte serum-free medium (SFM) with supplements including EGF (Epidermal Growth Factor) and Bovine Pituitary Extract (BPE), penicillin-streptomycin, trypsin, Dulbecco’s Phosphate Buffered Saline (DPBS) and Sodium pyruvate were purchased from Gibco (ThermoFisher, Cramlington, UK). Y-27632, Rho kinase inhibitor, and Sulforhodamine B (SRB) assay kit were purchased from Abcam, UK. Dimethyl sulfoxide (DMSO) and foetal bovine serum (FBS) were purchased from Sigma, UK. The GenElute RNA/DNA/Protein Purification Plus kit was purchased from Sigma-Aldrich, Gillingham, UK.

### 4.2. Collection and Purification of Whole F. carica L.

*F. carica* L. was collected drop by drop without squeezing over summer months from unripe fruits of fig trees in the suburb of Antalya, Turkey. We performed the purification of whole fig latex, as described in our previous study [25]. Briefly, the latex was initially filtered using a Whatman No. 1 filter from Fisher Scientific, UK. After filtration, it was then centrifuged at 13,000 rpm and a temperature of 4 °C to separate the polymeric gum from the liquid filtrate. The aqueous part was further purified via filtration using a disposable filter membrane with a pore size of 5 µm from Sigma, UK. It was stored at −20 °C for further analysis. The chemical analysis of the fig latex extract was conducted using established methodologies described in [25], forming the basis for our experimental approach to ensure consistency and reproducibility. NMR techniques (1 H, 13C, 1 H-1 H COSY, 1 H-1 H TOCSY, and 1 H-13C HSQC) were employed to identify and characterize components. Mass spectrometry analyses aligned with these methods were used for molecular elucidation. Additionally, HPLC techniques aided in the separation and identification of lipid species within the extract. The latex used in this study is the same as characterized in our previous research [25].

### 4.3. Cell Lines and Cell Culture Conditions 

HPV-positive human cervical cancer cell lines, specifically CaSki with HPV type 16 and HeLa with HPV type 18, were procured from the American Type Culture Collection (Manassas, VA, USA). HPV-negative human cervical keratinocytes, known as HCKT1 and generously provided by Prof. Tohru Kiyono from the Japan National Cancer Center, were also included in the study. To sustain the growth of HeLa and CaSki cells, a culture medium of DMEM supplemented with 10% heat-inactivated foetal bovine serum (FBS) and 100 μg/mL penicillin-streptomycin was employed. In contrast, HCKT1 cells were nurtured in a serum-free medium supplemented with 20 μg/mL BPE, 0.2 ng/mL EGF, and 10 μM Y-27632. All cell lines were cultivated in a controlled environment with 5% CO_2_ at 37 °C to maintain optimal humidity and conditions.

### 4.4. SRB Cell Viability Assay 

In order to investigate the effect of whole fig latex on cell growth, a Sulforodamine B (SRB) assay was performed. For cell viability analysis, the aqueous part of the plant extract was subjected to freeze-drying to obtain a powder form. The freeze-dried powder was then dissolved in DMSO to prepare a 1 mg/mL stock solution. Several concentrations were prepared by diluting the stock solution with cell culture medium. Human cervical cancer cells (HeLa and CaSki) and normal HCKT1 cells were cultured at a concentration of 5 × 10^4^ in 0.1 mL of medium, in a 96-well plate. On the following day, the cells were treated with various concentrations (5 μg/mL, 10 μg/mL, 50 μg/mL, 100 μg/mL, and 200 μg/mL) of fig latex. This selection of concentrations was based on preliminary dose–response experiments and previous research to identify concentrations exhibiting selective cytotoxicity towards HPV-positive cervical cancer cells while preserving normal cell viability [25]. After 72 h of treatment, the cells were fixed with fixation solution for 1 h. After 3 washes with distilled water, the cells were stained with SRB solution for 15 min and rinsed with washing solution 3 times. Protein-bound dye was solubilized, and the optical density was determined at 545 based on the manufacturer’s recommendations. For all experiments, the percentage of cytotoxicity was calculated as: [(O.D. vehicle) × (O.D. sample)/O.D. vehicle] × 100. Background correction was carried out by subtracting the O.D. of culture media. The percent of proliferation in each treated cell line was normalised based on their control wells. All experiments were performed at least in triplicate. All treatments were adjusted to equal concentrations of DMSO in the range 0.1~0.2%.

### 4.5. Cell Cycle Analysis 

Cell cycle distribution was assessed via flow cytometry. Cells were treated with 100 μg/mL of fig latex or equivalent amount of PBS for 72 h. Approximately (1 × 106) cells were harvested from both control and treated flasks. The cells were then washed in PBS and fixed in 70% ice-cold ethanol for 1 h. Then, 500 µL of PI/RNase (Thermofisher, UK) was added to the samples and they were kept in the dark for 20 min at 37 °C. The stained cells were then excited at 488 nm using the FL-3 detector (620 nM) of a BD FACs Calibur flow cytometer (Becton-Dickinson, Franklin Lakes, NJ, USA). The acquired data were analysed using CellQuest software (version 5.1) (Becton-Dickinson).

### 4.6. RNA Preparation 

Total RNA extraction from fig latex treated and untreated cell lines was performed using the Gen Elute kit (Sigma Aldrich, St. Louis, MO, USA) according to the manufacturer’s instructions. The quality of total RNA was assessed using The Agilent 2100 bioanalyzer (Agilent, Palo Alto, CA, USA) with RNA 6000 Nano LabChip kit (Agilent, Waldbronn, Germany). All RNA samples selected for sequencing had a RIN value greater than 7.5.

### 4.7. RNA Sequencing (RNA-Seq)

The RNA samples were sent to CeGaT GmbH, Germany, for library preparation, sequencing, and bioinformatic analysis. Libraries were prepared using the SMART-Seq Stranded Kit (Takara, Kusatsu, Japan). Multiplexed libraries were sequenced on the Illumina NovaSeq 6000 platform, at 100 bp paired end reads. The sequencing depth for each sample was >20 million reads. All samples passed quality control based on the manufacturer’s standards. RNA-Seq was performed in triplicate, and Universal Human Reference RNA (UHRR) was used as positive control to assess the quality and performance of the sequencing process.

### 4.8. Bioinformatic Analysis 

The sequence reads were analysed further by using diverse bioinformatic tools. The demultiplexing of the sequencing reads was performed with Illumina bcl2fastq (vs 2.20). Adapters were trimmed with Skewer (vs 0.2.2) [82]. The trimmed raw reads were aligned to hg19-cegat using STAR (version 2.7.3) [83]. Pseudoautosomal regions (PAR) were masked on chromosome Y (chrY:10001-2649520, chrY:59034050-59363566). Reads originating from these regions can be found at the respective location on chromosome X. Normalized counts have been calculated with DESeq2 (version 1.24.0) in R (version 3.6.1) [84]. DESeq2 uses a negative binomial generalized linear model to test for differential expression based on gene counts. 

For the functional enrichment analysis, the RNA sequencing (RNA-Seq) data obtained from drug-treated and untreated cells were used. Gene Set Enrichment Analysis (GSEA) was performed using the GSEA software (version 4.3.0) [85,86]. The RNA-Seq data sets were preprocessed and normalized, and the resulting gene expression profiles were analysed against a comprehensive collection of gene sets derived from public databases, such as MSigDB [85]. The GSEA algorithm computed an enrichment score for each gene set, indicating the extent to which the gene set was overrepresented among the differentially expressed genes. 

Moreover, EnrichR, an online platform for comprehensive gene set enrichment analysis, was utilized [87,88,89]. The preprocessed RNA-Seq data sets were uploaded to EnrichR, and the analysis was conducted by following the provided instructions. EnrichR integrates multiple pathway and gene set databases, such as KEGG and Reactome, to identify enriched pathways associated with the differentially expressed genes. The analysis generated enriched pathway results with corresponding statistical significance. The results obtained from both GSEA and EnrichR (version 3.2) were used to gain insights into the biological processes and pathways affected by the drug treatment in the cells [85,86,87,88,89]. The kinase enrichment analysis was performed by using Expression2 Kinases (X2K) software (version 0.0.4) [90,91]. 

### 4.9. Statistical Analysis 

The data were collected from at least three independent experiments and presented as the mean ± standard deviation for each group. Statistical analyses, including one-way analysis of variance (ANOVA) followed by post hoc Tukey’s test, were conducted using R Studio software with the ‘stats’ package for ANOVA and the ‘agricolae’ package for post hoc testing. A significance level of *p* < 0.05 was considered to indicate a statistically significant difference.

## 5. Conclusions

In this study, we explored the potential of fig latex as a therapeutic intervention for HPV-positive cervical cancer. Our findings demonstrate that fig latex selectively inhibits the growth of HPV-positive cancer cells, with no adverse effects on normal cells. This targeted effect suggests its potential as a novel treatment strategy. Additionally, fig latex induces cell cycle arrest in HPV-positive cells, further emphasizing its anti-cancer properties. Through a comprehensive RNA-Seq analysis, we unveiled significant alterations in gene expression profiles and identified key pathways affected by fig latex treatment. These pathways, including Nonsense-Mediated Decay, Cell Cycle regulation, and Transcriptional Regulation by TP53, shed light on the intricate molecular mechanisms underlying fig latex’s action against cervical cancer. Moreover, the identification of specific transcription factors and regulatory kinases affected by fig latex provides valuable insights into its potential as a precision-driven therapy. By modulating essential proteins involved in cell cycle regulation, fig latex demonstrates promise in disrupting critical pathways associated with cancer cell proliferation.

Overall, our study highlights fig latex as a promising natural compound with the potential to revolutionize treatment approaches for HPV-positive cervical cancer. Further research and clinical trials are warranted to fully harness the therapeutic benefits of fig latex in the fight against this formidable disease.

## Figures and Tables

**Table 1:** Pathway enrichment analysis of common genes in HPV-positive cervical cancer cell lines after fig latex treatment. The table includes the description of the biological pathway or process, the number of overlap genes from differentially expressed genes, the *p*-value, FDR q-value, and the specific genes that overlap for each pathway or process.

Description	Number of Overlap Genes	*p*-Value	FDR q-Value	Overlap Genes
Nonsense-Mediated Decay (NMD)	7	1.45 × 10^−7^	2.21 × 10^−4^	RPS27A, RNF111, RPS6, RPL27, RPL37, RPL39, UPF2, SMG6
Cell Cycle	12	5.91 × 10^−6^	3.02 × 10^−3^	PCNA, POLD3, PRIM1, ORC2, RAD1, YWHAQ, CDC14A, CENPK, CEP72, MZT1, DIDO1
Transcriptional Regulation by TP53	8	3.42 × 10^−5^	5.82 × 10^−3^	RPS27ARAD1, YWHAQ, RPS27A, PCNA, COX7A2L, NDRG1, PIP4K2B
Apoptotic Process	18	1.06 × 10^−4^	1.3 × 10^−2^	CUL5, DDRGK1, YEATS4, MORF4L2, KMT2A, PDCD6, HIF1A, CALR, CTSV, DNASE1, CHD8, MRPS30, RPS6, KLF4, PTMA, PHLPP1, DIDO1, EBAG9

**Table 2:** Analysis of transcription factors and kinases that regulate downregulated genes upon fig latex treatment. (**A**) The top transcription factors. (**B**) The top three predicted regulatory kinases. The predictions are ranked based on their combined statistical score (*p*-value).

(A)	
Transcription Factors	*p*-Value
NFYB	1.74 × 10^−6^
NFYA	8.53 × 10^−6^
MAX	2.23 × 10^−5^
**(B)**	
**Regulatory Kinases**	***p*** **-Value**
CDK4	6.74 × 10^−18^
CDK1	2.62 × 10^−16^
MAX	3.68 × 10^−14^

## Data Availability

Data are contained within the article and Supplementary Materials.

## Supplementary Materials

The following supporting information can be downloaded at: https://www.mdpi.com/article/10.3390/ph16121723/s1. Table S1: Differentially expressed genes following to fig latex treatment.
